# Integrated Metagenomic and Metabolomic Profiling of Boar Semen During Ambient-Temperature Storage

**DOI:** 10.3390/microorganisms14030560

**Published:** 2026-03-01

**Authors:** Haoshi Cheng, Jinyi Han, Kaiyuan Liu, Li Wang, Qiuyu Meng, Chuang Liu, Xuanjun Liu, Mingyu Wang, Feng Yang, Xinjian Li

**Affiliations:** 1 College of Animal Science and Technology, Henan Agricultural University, Zhengzhou 450046, China; 18837401070@163.com (H.C.); hjy7jane@126.com (J.H.); 15236388763@163.com (K.L.); 18338155530@163.com (L.W.); 13629929273@163.com (Q.M.); liuchuang9828@163.com (C.L.); 15137957782@163.com (X.L.); 2 Institute of Animal Science and Veterinary Medicine, Hainan Academy of Agricultural Sciences, Haikou 571100, China; wangmingyv623@163.com

**Keywords:** boar semen, ambient-temperature storage, 16S rRNA, metabolomics, multi-omics integration

## Abstract

The reproductive efficiency of breeding boars substantially influences swine industry productivity. Sperm viability during ambient-temperature storage is critically affected by environmental factors, including microbial activity. This study aimed to elucidate the dynamics and interactions between the seminal microbiome and metabolome during boar semen storage at 17 °C. Using integrated 16S rRNA sequencing and untargeted metabolomics, we analyzed semen samples from six healthy boars (31–33 months old) collected at day 0 (control), 2, 4, and 6 of storage. Our results demonstrate that storage leads to a marked decline in microbial diversity, progressive enrichment of the opportunistic genus *Proteus*, depletion of key antioxidant and cofactor metabolites such as vitamin B6, and extensive metabolic reprogramming—including alterations in short-chain fatty acid, purine, and lipid oxidation pathways. Multi-omics correlation analysis further revealed strong associations between microbial succession and metabolic shifts, highlighting their combined role in driving sperm functional decline. These findings provide a mechanistic basis for improving semen preservation strategies through microbiome and metabolite-targeted interventions.

## 1. Introduction

Porcine semen experiences a progressive decline in quality during storage at ambient temperature. This deterioration is governed by intricate bidirectional interactions between sperm metabolic state and the seminal microbiota—mediated through energy metabolism efficiency, redox balance, and the compositional and functional dynamics of the microbial community. On one hand, microbial proliferation competes for nutrients, modifies the seminal microenvironment, and releases metabolites and reactive oxygen species (ROS), collectively aggravating metabolic stress and oxidative damage to spermatozoa. On the other hand, shifts in sperm metabolism and changes in the extender milieu reciprocally influence microbial succession [1]. Such metabolic–microbial crosstalk constitutes a central driver of declining sperm function over time.

As a nutrient-rich fluid, porcine semen is susceptible to bacterial colonization. The seminal bacteria can be broadly divided into beneficial and detrimental populations: probiotic species support semen quality maintenance, whereas pathogenic bacteria compromise sperm function and reproductive outcomes [2]. Seminal microbiota composition varies markedly among boar studs, and these structural differences correlate with key sperm quality parameters [3]. For example, elevated relative abundances of *Prevotella*, *Ruminococcus*, and *Bacteroides* are negatively associated with sperm motility. Furthermore, the seminal microbiome exhibits seasonal dynamics: Lactobacillus—more abundant in winter—correlates positively with sow fertility following artificial insemination, whereas summer-enriched Pseudomonas is linked to diminished sperm quality and reproductive potential [4]. Thus, a deeper understanding and targeted modulation of seminal microbial ecology are critical for enhancing semen preservation.

Metabolomics offers another essential lens for deciphering the molecular basis of semen preservability. Recent metabolomic investigations have elucidated metabolic foundations of normal reproductive physiology [5], reproductive pathology [6], sperm freezability [7], and semen quality variation [8,9]. In porcine seminal plasma, organic acids and lipids constitute major metabolite classes, and perturbations in amino acid metabolism appear pivotal in limiting ambient-temperature storage longevity [10]. Moreover, metabolites such as D-proline and arginine have emerged as potential biomarkers of boar semen storage potential. Consequently, systematic profiling and regulation of key metabolites during storage hold considerable promise. However, current studies remain largely confined to single-omics approaches, lacking integrative functional insights into metabolic–microbial interplay.

In summary, the dynamic interface between the seminal metabolome and microbiome during ambient-temperature storage, and its functional consequences for sperm physiology, constitutes a complex and underexplored biological problem. Here, we integrate 16S rRNA gene sequencing and untargeted metabolomics data from porcine semen across multiple storage time points to perform a combined multi-omics analysis. Our objectives are to identify microbial signatures and critical metabolic pathways linked to reproductive performance, and to delineate their mechanistic roles in sperm physiology. This work is expected to establish a theoretical foundation for extending boar semen storage and to propose novel intervention strategies—via microbial community modulation—to advance livestock semen preservation technologies.

## 2. Material and Methods

All animal experiments in this study were strictly conducted in compliance with the Guidelines for the Care and Use of Experimental Animals issued by the Ministry of Science and Technology of the People’s Republic of China (Approval No. DWLL20211193). The experimental protocols were reviewed and approved by the Animal Care and Use Committee of Henan Agricultural University, and all procedures involving animal handling, semen sample collection and preservation were strictly performed in accordance with the approved ethical norms and relevant regulatory standards for experimental animals. All pigs used in the study were privately raised, and verbal consent was obtained from the pig owners regarding semen sample collection prior to the conduct of sampling work.

### 2.1. Sample Collection and Processing

Semen was collected from six healthy, reproductively proven Duroc boars aged 31–33 months at a commercial stud using the gloved-hand technique. The experiment was performed from June to August 2023 (summer). Immediately after collection, each ejaculate was filtered through three layers of sterile gauze, placed into a pre-warmed thermal container, and transported to the laboratory via pneumatic tubing within 5 min for initial evaluation. Samples displaying normal appearance and odor, with progressive motility ≥80% and a morphological abnormality rate ≤ 20%, were selected for further processing.

Selected semen was diluted to a final concentration of 2.5 × 10^9^ spermatozoa per 80 mL using the HNAU extender (Henan Agricultural University, Zhengzhou, China) developed in this study, and stored at 17 °C in a temperature-controlled incubator. During storage, samples were gently mixed every 12 h. Sperm motility was assessed at 24 h intervals using a computer-assisted sperm analysis system (CASA, Minitube, Tiefenbach, Germany). At storage time points 0, 2, 4, and 6 days, 10 mL of thoroughly mixed semen was collected from each sample, snap-frozen in liquid nitrogen, and stored at −80 °C until analysis. All samples were processed simultaneously after the completion of the collection period.

The diluent used in this study (designated as HNAU extender) was prepared in detail as follows. A one-liter volume of semen extender was prepared containing 14.00 g fructose, 14.00 g glucose, 6.90 g sodium citrate, 2.35 g sodium EDTA, 1.00 g sodium bicarbonate, 2.90 g citric acid, 6 g Tris, 0.70 g potassium chloride, 7.50 g skim milk, 2.50 g bovine serum albumin, 7.50 g HEPES, and 10 mL of 100× penicillin–streptomycin–gentamicin solution (P1410, Solarbio, Beijing, China). The 100× penicillin–streptomycin–gentamicin solution was added at a final concentration of 1% (*v*/*v*) to inhibit the growth of bacteria and prevent microbial contamination during semen processing and storage. After preparation, the extender was filtered through a 0.2 μm PES syringe filter (SLGPR33RB, Millipore Express, Billerica, MA, USA) to further remove potential microorganisms.

According to storage duration, samples were categorized into four experimental groups: day 0 (control), day 2, day 4, and day 6. These groups were used for subsequent multi-omics analyses.

#### S rRNA Sequencing

Total genomic DNA was extracted from semen samples using a modified cetyltrimethylammonium bromide (CTAB) protocol. In brief, 1 mL of CTAB lysis buffer and an appropriate amount of lysozyme were added to each semen aliquot in a 2 mL microcentrifuge tube, followed by incubation at 65 °C. After lysis, the mixture was centrifuged and the supernatant was extracted with an equal volume of phenol: chloroform: isoamyl alcohol (25:24:1). The aqueous phase was then extracted with chloroform: isoamyl alcohol (24:1), and DNA was precipitated with 0.7 volumes of isopropanol at −20 °C for 30 min. The pellet was washed twice with 75% ethanol, air-dried, and dissolved in dd H_2_O (with incubation at 55–60 °C if needed). RNA was removed by incubation with 1 µL of RNase A at 37 °C for 15 min.

DNA quality was verified spectrophotometrically and diluted to 1 ng/µL for subsequent amplification. The V3–V4 hypervariable regions of the bacterial 16S rRNA gene were amplified by PCR using the primers 341F (5′-CCTAYGGGRBGCASCAG-3′) and 806R (5′-GGACTACNNGGGTATCTAAT-3′). The thermal cycling conditions consisted of initial denaturation at 98 °C for 1 min; 30 cycles of denaturation at 98 °C for 10 s, annealing at 50 °C for 30 s, and extension at 72 °C for 30 s; followed by a final extension at 72 °C for 5 min. Amplification products were analyzed on a 2% agarose gel in 1× TAE buffer. Bands of the expected size were excised and purified using a commercial DNA purification kit (TIANGEN). Purified amplicons were quantified with the Quanti Fluor™-ST system (Promega) and used to construct sequencing libraries following the standard Illumina MiSeq PE2 × 300 workflow (TIANGEN Biotech (Beijing) Co., Ltd., Beijing, China). Paired-end sequencing (2 × 300 bp) was performed on the Illumina MiSeq platform.

### 2.2. Non-Targeted Metabolome Sequencing

A 100 µL aliquot of each sample was mixed with 400 µL of ice-cold 80% methanol in a 1.5 mL microcentrifuge tube, vortexed thoroughly, and incubated on ice for 5 min. After centrifugation at 15,000× *g* for 20 min at 4 °C, a portion of the supernatant was diluted with MS-grade water to a final methanol concentration of 53%, and centrifuged again under identical conditions. The resulting supernatant was used for liquid chromatography–mass spectrometry (LC–MS) analysis.

Raw LC–MS data (.raw files) were processed using Compound Discoverer 3.1. Initial metabolite screening was performed based on retention time and mass-to-charge ratio, followed by peak alignment (retention time tolerance = 0.2 min, mass deviation = 5 ppm). Peaks were extracted and quantified using the following criteria: mass deviation ≤ 5 ppm, signal intensity variation ≤ 30%, signal-to-noise ratio ≥ 3, along with consideration of minimum intensity thresholds and adduct ion patterns. Molecular formulas were predicted from molecular ion peaks and fragmentation patterns and matched against the mzCloud, mzVault, and Masslist databases. Background signals were subtracted using blank samples, and raw quantification data were normalized to yield identified metabolites with relative abundances.

All computational analyses were conducted under a Linux environment (CentOS 6.6) using R and Python 3.8.x as the primary programming languages.

### 2.3. Data Processing

Microbial sequencing data were processed entirely in QIIME2 (v1.8.0). Primers were trimmed from paired-end reads using qiime cutadapt trim-paired, and reads lacking primer matches were discarded. Denoising, merging, and chimera removal were performed with the DADA2 plugin (qiime dada2 denoise-paired). After processing all samples, amplicon sequence variant (ASV) tables and representative sequences were merged, and singleton ASVs were filtered out.

Taxonomic assignment of ASVs was conducted against the Greengenes database (v13.8) using the qiime phylogeny align-to-tree-mafft-fasttree pipeline, which aligns sequences with MAFFT and infers a phylogenetic tree with Fast Tree. An ASV abundance table was generated and rarefied to an even sequencing depth across samples using qiime feature-table rarefy to enable standardized comparative analyses. Metabolites and microbial features were functionally annotated using the KEGG, HMDB, and LIPIDMaps databases, respectively.

All other phenotypic analyses and visualizations were performed in R (v4.5.0). Boar semen quality parameters across storage time points are reported as mean ± standard deviation, with statistical significance defined as *p* < 0.05.

## 3. Results

### 3.1. The Influence of Different Storage Times on Semen Motility

During ambient-temperature storage, semen motility exhibited a progressive, time-dependent decline (Table 1). Initial motility (day 0) was significantly higher than at all subsequent time points (days 1–6; *p* < 0.05). By day 6, motility had further decreased and was significantly lower than at every preceding measurement (*p* < 0.05). Similarly, the proportion of progressively motile sperm peaked on day 0—significantly exceeding values from days 2–6 (*p* < 0.05)—and reached its lowest level on day 6, differing significantly from all earlier time points (*p* < 0.05).

Kinematic parameters followed a comparable trajectory. Both curvilinear velocity (VCL) and straight-line velocity (VSL) remained elevated through the first day of storage but dropped markedly from days 3–4 onward. By day 6, VCL and VSL were significantly reduced relative to values observed during days 0–5 (*p* < 0.05). Average path velocity (VAP) also declined gradually over time, with significantly higher values on days 0–1 compared to days 3–6 (*p* < 0.05); VAP was lowest at day 6.

Collectively, all measured motility parameters deteriorated with extended storage, with a pronounced acceleration in decline beyond day 4, indicating a critical threshold for semen quality maintenance under ambient-temperature conditions.

### 3.2. Dynamic Changes in the Semen Microbial Community Structure

**Figure 1:**
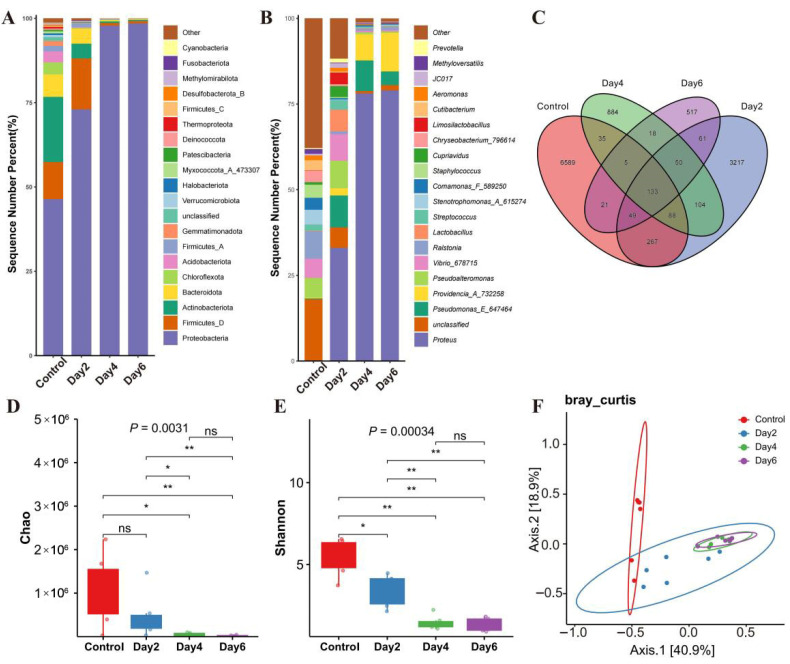
Microbial composition and diversity in semen during ambient-temperature storage. (**A**,**B**) Taxonomic composition at the phylum (**A**) and genus (**B**) levels, shown as relative abundance. (**C**) Venn diagram of Amplicon Sequence Variants (ASVs) shared among storage time points. (**D**) Box plot of the Chao1 index, an estimator of microbial richness that accounts for undetected rare species. (**E**) Box plot of the Shannon index, reflecting both richness and evenness of the microbial community. (**F**) Principal coordinates analysis (PCoA) of the seminal microbiota across storage durations. (* *p* < 0.05, ** *p* < 0.01; ns, no significant difference.)

Taxonomic profiling across the four storage time points identified 57 phyla, 165 classes, 321 orders, 556 families, and 1150 genera (Figure 1A,B). At the phylum level, the seminal microbiota was dominated by *Proteobacteria*, *Firmicutes_D*, *Actinobacteria*, *Bacteroidota*, and *Chloroflexi*. Among these, the relative abundance of *Proteobacteria* increased significantly over storage time. Concordantly, at the genus level, *Proteus* emerged as the most abundant taxon and exhibited a similar time-dependent rise in abundance. Comparison of Amplicon Sequence Variants (ASVs) revealed a progressive loss of microbial richness with extended storage (Figure 1C). A total of 12,038 ASVs were detected across all samples. The number of ASVs declined markedly from 7187 in the control group (day 0) to 3969 (day 2), 1317 (day 4), and 854 (day 6), with only 133 ASVs shared among all time points.

Alpha diversity indices corroborated this decline. Both the Chao1 and Shannon indices decreased significantly over time (Figure 1D,E), indicating a sustained reduction in microbial richness and evenness. Notably, diversity metrics did not differ significantly between days 4 and 6, suggesting stabilization of the community structure after four days of storage. Beta diversity analysis (Figure 1F) further demonstrated temporal restructuring of the microbiota. The control group clearly separated from all stored samples in ordination space. In contrast, communities from days 2, 4, and 6 gradually converged, with substantial overlap observed between days 4 and 6, indicating increasingly similar composition profiles over time.

In summary, ambient-temperature storage drove a pronounced decline in microbial diversity alongside a progressive enrichment of specific taxa—most notably Proteobacteria and its constituent genus *Proteus*. These shifts reflect a directional succession of the seminal microbiota during storage, characterized by reduced complexity and increased dominance of a subset of bacterial lineages.

### 3.3. Differential Microbial Abundance and Interactions in Semen

**Figure 2:**
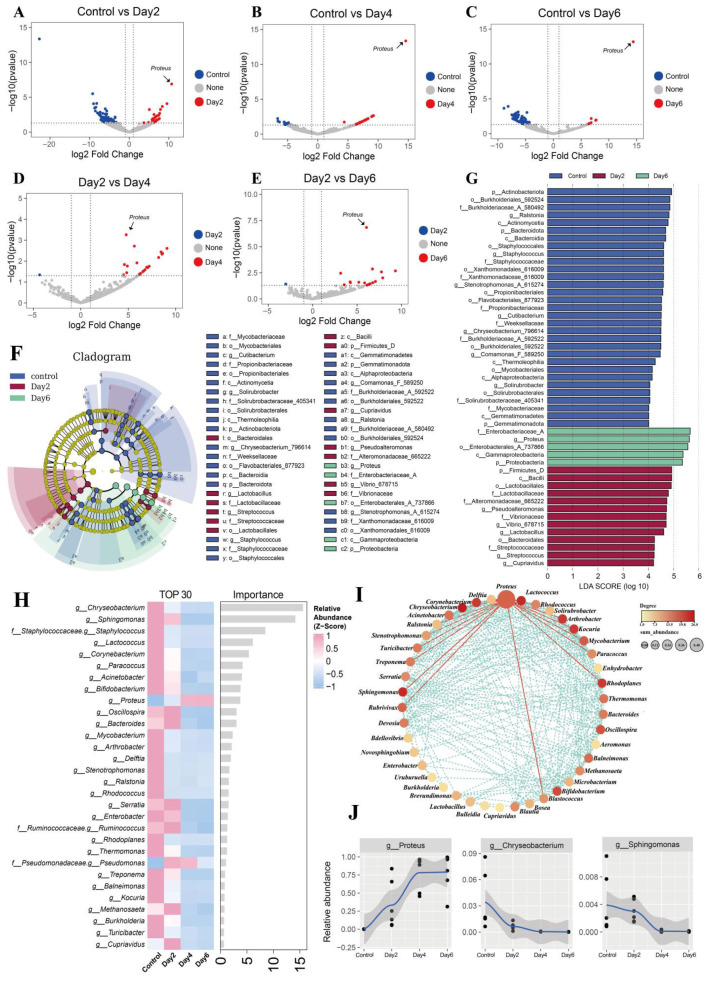
Differential microbial abundance and interaction networks across storage time points. (**A**–**E**) Volcano plots comparing microbial abundance between: Control vs. Day 2 (**A**), Control vs. Day 4 (**B**), Control vs. Day 6 (**C**), Day 2 vs. Day 4 (**D**), and Day 2 vs. Day 6 (**E**). (**F**) Cladogram illustrating phylogenetic distribution of differentially abundant taxa. (**G**) Histogram of LDA scores (LDA > 4) highlighting taxa that most strongly discriminate storage groups. (**H**) Random forest classification identifying microbial genera important for distinguishing time points. (**I**) Co-occurrence network at the genus level. (**J**) Line plots showing abundance dynamics of selected differential genera over storage time.

To characterize storage-associated shifts in the seminal microbiota, we performed differential abundance analysis using DESeq2 and LEfSe. Substantial differences in microbial composition were observed between time points (Figure 2A–E). Compared with the control (day 0), day 2, day 4, and day 6 exhibited 116, 65, and 85 differentially abundant genera, respectively. Between day 2 and day 4, 65 genera differed significantly, while only 21 genera differed between day 2 and day 6. No significant differential abundance was detected between day 4 and day 6, consistent with the alpha-diversity stabilization noted earlier. Notably, *Proteus* was the only genus consistently differentially abundant across all comparisons. LEfSe identified 49 taxonomic clades exhibiting significant variation across groups (LDA ≥ 5, *p* < 0.001), spanning 5 phyla, 7 classes, 11 orders, 13 families, and 13 genera (Figure 2F,G). These clades were primarily distributed among the control, day 2, and day 6 groups, with *Proteobacteria* (genus *Proteus*) showing marked enrichment in the day 6 samples. Random forest analysis ranked bacterial genera by their importance in distinguishing storage time points (Figure 2H). *Chryseobacterium* emerged as the most influential taxon (IncNodePurity = 15.57), followed by *Sphingomonas* (14.12). *Proteus* ranked ninth in importance (3.69) and represented the most abundant genus among the top 50 contributors; it was also the only genus whose relative abundance increased consistently during storage.

Correlation analysis among the top 50 genera (|r| > 0.65, *p* < 0.05) revealed a network of 42 significantly associated taxa (Figure 2I,J). Within this network, *Proteus* showed strong negative correlations with 17 other genera—including *Chryseobacterium*, *Lactococcus*, and *Ralstonia*—highlighting its antagonistic relationship with multiple resident community members during storage.

### 3.4. Functional Profiling of the Seminal Microbiota

**Figure 3:**
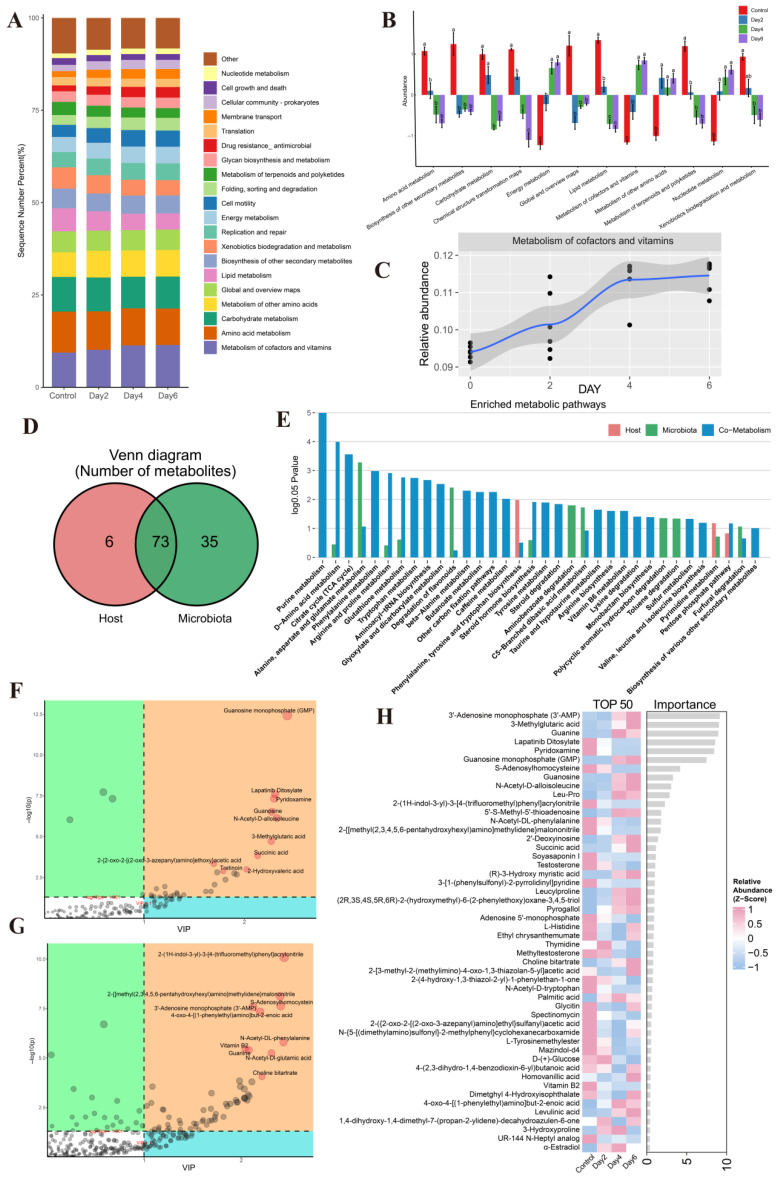
Functional analysis of the seminal microbiota and composition/differential analysis of metabolites across storage time points. (**A**) Predicted microbial metabolic functions based on KEGG pathways. (**B**) Temporal trends of significantly altered microbial functional pathways during storage, Different lowercase letters (a, b, c) represent significant differences between groups at the same storage time point (*p* < 0.05). (**C**) Line plot showing changes in metabolism of cofactors and vitamins over time. (**D**) Venn diagram illustrating the origins of detected metabolites (host-derived, microbial-derived, or shared). (**E**) KEGG pathway enrichment analysis of metabolites grouped by origin. (**F**,**G**) Orthogonal partial least-squares discriminant analysis (OPLS-DA) score plots in negative-ion (**F**) and combined positive-/negative-ion (**G**) modes. (**H**) Random forest analysis ranking the most influential metabolites across storage intervals.

Functional analysis of the microbiota across storage time points revealed 47 KEGG level-2 pathways, with metabolism of cofactors and vitamins, amino acid metabolism, and carbohydrate metabolism representing the most abundant functional categories (Figure 3A). Among these, metabolism of cofactors and vitamins was the predominant function and showed a progressive increase from the control through day 4.

Comparative functional analysis identified 12 pathways that differed significantly between groups (Figure 3B). Both cofactor and vitamin metabolism and energy metabolism increased significantly with longer storage, although no further change was observed between days 4 and 6. This pattern aligns with the compositional stabilization of the microbiota after day 4, indicating that functional shifts parallel the structural convergence of the community.

Collectively, these results demonstrate that ambient-temperature storage drives marked functional remodeling of the seminal microbiota, characterized by a progressive enrichment of specific metabolic capacities—most notably in cofactor/vitamin and energy metabolism—until functional profiles stabilize after four days of storage.

### 3.5. Composition and Diversity of the Seminal Metabolome

Metabolomic profiling identified 310 cationic and 121 anionic metabolites across all samples. Annotation against the KEGG database assigned 144 functional roles, encompassing peptides, organic acids, vitamins and cofactors, nucleic acids, carbohydrates, steroids, lipids, antibiotics, hormones, and neurotransmitters. Tracing the origins of these metabolites revealed that 79 were host-derived, 108 were microbial-derived, and 73 originated from both sources (Figure 3D). Collectively, these metabolites were enriched in 35 KEGG pathways (Figure 3E). Purine metabolism was the most significantly enriched pathway, involving 11 metabolites. Pyridoxamine, pyridoxine, and D-erythrose-4-phosphate were notably enriched in vitamin B6 metabolism, whereas riboflavin metabolism—represented only by vitamin B2—did not reach statistical significance.

OPLS-DA showed clear separation between storage time points, and variable importance in projection (VIP) scores highlighted metabolites that contributed most to group discrimination (Figure 3F,G). Among these, 60 positive-ion and 30 negative-ion metabolites met the criteria of *p* < 0.05 and VIP > 1. Random forest analysis identified the top 50 most influential metabolites (Figure 3H). These included the vitamin/cofactor-related compounds pyridoxamine and vitamin B2, both of which declined progressively with storage time.

**Figure 4:**
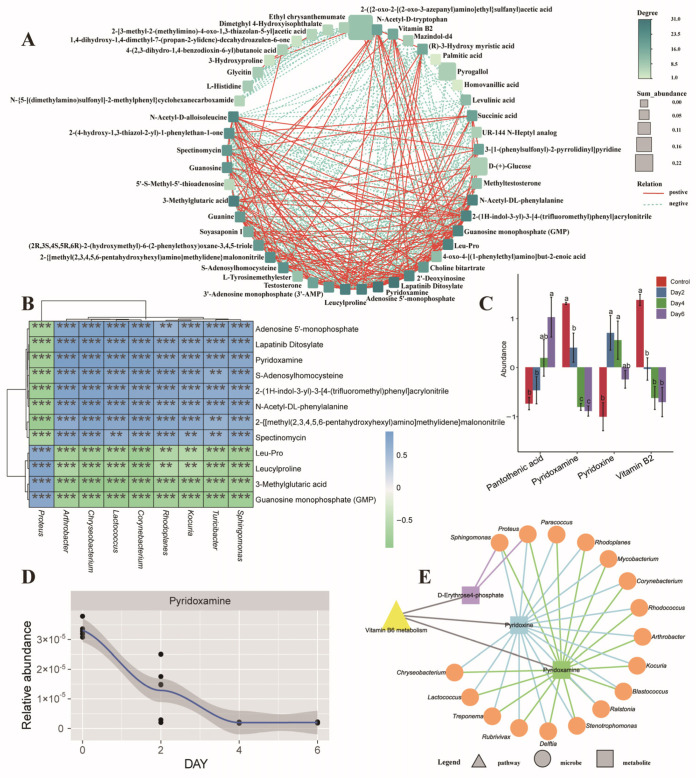
Integrated correlation analysis between the seminal microbiome and metabolome. (**A**) Co-occurrence network of the top 50 metabolites identified by random forest analysis. (**B**) Correlation heatmap between metabolites and microbial genera with degree values > 35,** *p* < 0.01, *** *p* < 0.001. (**C**) Temporal trends of the four vitamin- and cofactor-related metabolites that differed significantly among storage groups. (**D**) Line plot showing the abundance dynamics of pyridoxamine over storage time. (**E**) Interaction network linking key vitamin-B6-pathway metabolites with correlated microbial taxa.

Correlation network analysis of these top 50 metabolites revealed 48 significant interactions (|r| > 0.65, *p* < 0.05; Figure 4A). Lapatinib ditosylate and pyridoxamine emerged as the most connected metabolites (degree = 31 each), followed by vitamin B2 (degree = 17). Pyridoxamine and vitamin B2 exhibited a strong positive correlation (r ≈ 0.79, *p* < 0.05), underscoring coordinated changes in key vitamin-related metabolites during storage.

### 3.6. Integrative Analysis of the Seminal Microbiome and Metabolome

From the top 50 microbiota and metabolite features identified by random forest analysis, we constructed a correlation network comprising 41 microbial genera and 38 metabolites that showed strong associations (|r| > 0.65, *p* < 0.05). *Chryseobacterium* exhibited the highest connectivity (degree = 31), followed by *Proteus*, which correlated with 22 metabolites. Among metabolites, 3-methylglutaric acid was most highly connected (degree = 33), followed by pyridoxamine (degree = 28); vitamin B2 correlated with eight microbial taxa.

A focused heatmap of the top 20 nodes (degree > 35) revealed that *Proteus* correlated positively with Leu-Pro, leucylproline, 3-methylglutaric acid, and guanosine monophosphate, but negatively with pyridoxamine and related metabolites (Figure 4B).

Given the progressive enrichment of microbial cofactor and vitamin metabolism functions during storage, we next examined corresponding changes in vitamin- and cofactor-related metabolites. Of eight such metabolites detected in the seminal metabolome, four showed significant variation across storage time points: pyridoxine, vitamin B2, pantothenic acid, and pyridoxamine (Figure 4C). Among these, pyridoxine and vitamin B2 declined consistently over time (Figure 4C,D), mirroring the functional shift in microbial vitamin metabolism.

## 4. Discussion

Ambient-temperature liquid storage remains a cornerstone of modern swine reproduction, enabling widespread dissemination of superior genetics through artificial insemination. While current extenders maintain acceptable sperm viability and fertilizing capacity during short-term storage, there is a clear need to improve preservation protocols to extend usable shelf life and consistency [11]. Our integrated multi-omics analysis across four storage time points reveals a dynamic interplay between the seminal microbiota and metabolome during storage. We observed a pronounced decline in microbial diversity alongside the progressive enrichment of opportunistic taxa such as *Proteus*. Concomitantly, key vitamin-related metabolites—particularly those in the vitamin B6 pathway—declined, while microbial-associated small molecules, including specific organic acids and lipid oxidation products, underwent significant alteration.

The increase in sperm motility on day 2 and velocity parameters on day 1 of storage represents a short-term adaptive recovery of boar sperm after dilution. Fresh boar sperm experience temporary osmotic and mechanical stress upon dilution, leading to suppressed motion characteristics in the early stage. As sperm adapt to the optimized extender environment and recover from stress, motility and velocity show a transient improvement. This initial increase is a normal physiological adaptation rather than a permanent enhancement, and sperm quality gradually declines with prolonged storage.

Bacterial contamination of boar semen is well-documented, especially during extended liquid storage. Previous studies have reported increased oxidative stress after 86 h of storage at 15 °C [12] and identified bacterial overgrowth and community dysbiosis as major contributors to sperm quality decline [13]. Our findings of reduced microbial diversity and enrichment of opportunistic *Proteus* align with these reports and reinforce the view that bacterial proliferation drives functional deterioration in stored semen. Notably, *Proteus* spp. are frequently isolated from boar artificial insemination centers, with some strains showing antibiotic resistance [14], and their growth during storage has been correlated with loss of sperm membrane integrity and motility [15]. In our data, *Proteus* abundance correlated positively with metabolites such as 3-methylglutaric acid and GMP, but negatively with antioxidant vitamin-B6-related compounds. This is consistent with reports that *Proteus* mirabilis can damage sperm membranes and induce apoptosis via secreted outer-membrane vesicles [16] and protein-degrading enzymes [17], processes that likely contribute to the oxidative and membrane-related metabolic shifts we observed.

Antibiotic supplementation remains a common strategy to control bacterial growth in semen extenders. However, rising antimicrobial resistance—reported in over 58% of Gram-negative boar semen isolates for penicillin [18]—underscores the need for alternatives [19]. Promising non-antibiotic agents include antimicrobial peptides (e.g., A-11 and AP19), which effectively inhibit common pathogens while preserving sperm quality [20], and ε-poly-L-lysine, which at optimal concentrations reduces bacterial load and improves sperm motility and membrane integrity [21]. Such approaches may offer sustainable routes to mitigate microbial-driven damage without exacerbating resistance.

From a metabolic perspective, oxidative stress [22], cofactor availability [23], and nutrient balance [24] are recognized as critical factors in semen preservation. Exogenous antioxidants such as tocopheryl carboxylate can reduce lipid peroxidation and enhance antioxidant enzyme activities during storage [25], and supplements like CoQ10, L-proline, and glycine derivatives have shown beneficial effects [26,27,28,29]. Here, we found that vitamin-B6-related metabolites (pyridoxamine, pyridoxal) declined progressively alongside the rise in *Proteus*, suggesting that VB6 depletion may represent a previously overlooked metabolic vulnerability during storage. Although VB6 supplementation has not been explicitly tested in boar semen extenders, its role in supporting antioxidant defenses and gonadal function in male reproduction is well established [30,31].

Most prior studies linking microbes and metabolites in boar reproduction have focused on the gut-testis axis. Investigations directly examining microbial-metabolite crosstalk within semen during storage are scarce. Emerging evidence suggests that microbial metabolic activity—rather than mere abundance—may drive functional outcomes through the production of secondary metabolites (e.g., short-chain fatty acids, bile-acid-like compounds, and amino-acid degradation products) that interact with spermatozoa [32]. Interestingly, reduced storage temperature (5 °C) can suppress the exponential growth of multi-drug-resistant bacteria and attenuate their damaging effects [33]. Our data indicate that during ambient-temperature storage, the seminal microbiota shifts from a mutualistic, low-activity state toward a metabolically active, opportunistic profile. This transition likely accelerates sperm damage through the release of oxidants, enzymes, toxins, and altered local metabolite pools—including the depletion of VB6 metabolites. Strikingly, a recent gut-semen multi-omics study also identified vitamin-B6 metabolism, along with short-chain fatty acid and bile-acid biosynthesis, as key pathways through which the microbiota modulates semen quality via the metabolite-host axis [34]. Our work extends this concept by demonstrating that similar microbial-metabolic dynamics operate within the seminal compartment itself during storage, offering new targets for intervention aimed at prolonging semen viability. 

## 5. Conclusions

By integrating 16S rRNA sequencing and untargeted metabolomics, this study reveals the dynamic interplay between the seminal microbiome and metabolome during ambient-temperature storage and its impact on boar semen quality. We demonstrate that storage is accompanied by a marked decline in microbial diversity, progressive enrichment of the opportunistic genus *Proteus*, depletion of key antioxidant and cofactor metabolites such as vitamin B6, and extensive metabolic reprogramming—including alterations in short-chain fatty acid, purine, and lipid oxidation pathways. These interconnected shifts collectively drive the functional deterioration of sperm over time. Our findings provide a mechanistic framework for understanding the limitations of current semen preservation protocols and highlight potential targets for intervention. Specifically, modulating the seminal microbial community or supplementing depleted metabolic cofactors—such as vitamin B6—may offer novel strategies to extend the functional longevity of stored boar semen. This work thus advances the prospects for microbiome-informed approaches to improve livestock reproduction and semen preservation technologies.

## Figures and Tables

**Table 1:** Comparison of semen quality parameters following different storage times.

	0 Day	1 Day	2 Day	3 Day	4 Day	5 Day	6 Day
Sperm Motility (%)	82.88 ± 4.64 ^a^	70.18 ± 5.98 ^bc^	74.26 ± 6.05 ^ab^	65.60 ± 4.82 ^bc^	62.73 ± 3.50 ^cd^	63.95 ± 9.02 ^bc^	51.03 ± 3.98 ^e^
Progressive Sperm Motility (%)	71.46 ± 8.26 ^a^	64.11 ± 6.23 ^ab^	59.48 ± 5.20 ^b^	52.49 ± 8.29 ^bc^	48.87 ± 6.20 ^c^	45.74 ± 5.98 ^c^	38.88 ± 5.08 ^d^
Curvilinear Velocity (μm/s)	81.18 ± 17.01 ^ab^	85.75 ± 17.12 ^a^	68.88 ± 7.44 ^ab^	62.81 ± 9.67 ^bc^	59.36 ± 6.14 ^bc^	57.83 ± 4.91 ^b^	49.37 ± 4.03 ^c^
Straight-Line Velocity (μm/s)	25.81 ± 6.51 ^ab^	28.43 ± 5.54 ^a^	19.53 ± 2.98 ^bc^	17.99 ± 3.76 ^bc^	18.46 ± 5.61	18.36 ± 7.51 ^ab^	15.60 ± 6.38 ^c^
Average Path Velocity (μm/s)	37.45 ± 8.56 ^ab^	41.04 ± 8.46 ^a^	30.35 ± 4.32 ^b^	27.76 ± 6.64 ^bc^	28.19 ± 6.85 ^bc^	25.75 ± 4.06 ^b^	21.89 ± 3.45 ^c^

Note: Within the same row, values with different superscript letters are significantly different (*p* < 0.05), while values sharing a common superscript letter or without a superscript letter do not differ significantly (*p* ≥ 0.05).

## Data Availability

The original contributions presented in this study are included in the article. Further inquiries can be directed to the corresponding authors.
